# Getting a consensus: advantages and disadvantages of Sepsis 3 in the context of middle-income settings

**DOI:** 10.5935/0103-507X.20160068

**Published:** 2016

**Authors:** Flavia Ribeiro Machado, Murillo Santucci Cesar de Assunção, Alexandre Biasi Cavalcanti, André Miguel Japiassú, Luciano Cesar Pontes de Azevedo, Mirella Cristine Oliveira

**Affiliations:** 1 Instituto Latino Americano de Sepse - São Paulo (SP), Brazil.; 2 Associação de Medicina Intensiva Brasileira - São Paulo (SP), Brazil.

## What is new on the Sepsis 3 definitions?

Recently the Society of Critical Care Medicine (SCCM) and the European Society of Critical Care Medicine (ESICM) promoted a new consensus conference and published the new sepsis definitions, known as Sepsis 3.^(1)^

Briefly, the broad definition of sepsis is now "a life-threatening organ dysfunction caused by dysregulated host response to infection".^(1)^ The clinical diagnosis of organ dysfunction is based on a variation of 2 or more points in the Sequential (Sepsis-related) Organ Assessment Score (SOFA). The presence of systemic inflammatory response syndrome (SIRS) criteria is no longer required for the definition. One of the main messages is that all sepsis should be considered as a severe disease so the term "severe sepsis" was abolished. Septic shock is defined as "a subset of sepsis with particularly profound circulatory, cellular and metabolic abnormalities associated with a greater risk of mortality than sepsis alone". The diagnostic criteria of septic shock are "vasopressor requirement required to maintain a mean arterial pressure of > 65mmHg and a serum lactate level > 2mmol/L in the absence of hypovolemia".^(2)^

In addition, the task force suggested the use of a simplified SOFA score, named quick SOFA, or qSOFA as a bedside tool to rapidly identify adult patients more likely to have poor outcomes if they have infection. So it's only a screening tool for identify critically ill patients and it does not define sepsis. qSOFA gives an alarm that means "don't loose time, if you haven't done anything yet, please act now". qSOFA is positive if the patient has at least 2 of the following clinical criteria: respiratory rate of 22/min or greater, altered mentation (Glasgow Coma Scale of < 15) or a systolic blood pressure of 100mmHg or less.^(3)^

Although the definitions have been endorsed by many societies of critical care around the world, they have also generated a lot of controversy mainly related to the increase in specificity at expense of reducing sensitivity. Thus, our aim in this document is to point out the main advantages as well as the disadvantages of the new definitions in the context of our country. We also aim to build up a consensus on how these new concepts should be applied in our daily practices, focusing in the quality improvement programs that are the key stone in our efforts to reduce sepsis mortality rates in Brazil,^(4)^ which are currently unacceptable.

## The advantages

The new definitions brought several improvements to the sepsis field. First, the broad definition of sepsis as the presence of organ dysfunction due to dysregulated response to infection was welcome as the previous notion of sepsis as a pure inflammatory host response is no longer physiologically plausible.^(5)^ The key issue in an infection process is the presence of the infectious agent who is, by itself, able to damage tissue and to lead to death. Both inflammation and immunosuppression are present as part of the response to the microorganism. Therefore, the term "dysregulated host response" is more adequate to describe the pathophysiological process.

Second, for the first time, the consensus was based on available data and not on expert opinion. The definition of sepsis, or better saying, the definition of organ dysfunction, was based on the predictive validity for death or prolonged intensive care unit (ICU) stay. The task force used data from 3 large US and one German database to select the best score. They tested the most known organ dysfunction scores, such as SOFA and a modified version of the Logistic Organ Dysfunction System (LODS) score.^(3)^ Although most of the studies used similar definitions of organ dysfunction as those used by the Surviving Sepsis Campaign quality improvement program, there was some degree of variation that might have compromised consistence. The standardization of the organ dysfunction criteria might help the inclusion of similar patients in future clinical trials as well as in epidemiological studies. It is also possible that using variation in the score rather than the score itself will better account for previous chronic dysfunctions.

Third, the new definitions do not require the presence of SIRS. Systemic inflammatory response syndrome is neither sensitive nor specific for sepsis. At least one in eight critically ill patients with sepsis do not develop SIRS criteria^(6)^ and up to 47% of hospitalized patients in general wards develop 2 SIRS criteria during their hospital stay.^(7)^ Although SIRS criteria remain of upmost relevance as a screening tool for potentially infected patients, particularly in the context of quality improvement programs, they are not fundamental to define the presence of sepsis.

Fourth, the nomenclature simplification: no more "severe" sepsis but rather only "sepsis". Over time, this will be an important shift to enhance the association of the word sepsis with a serious condition in terms of promoting better understanding of sepsis by health professionals and lay people. In other words, sepsis is always severe according with the new definition.

Fifth, although we have concerns about the specific criteria used to defined septic shock discussed latter, the new septic shock broad definition represents an advance in terms of the 1992 consensus that did not define properly what the state of shock means. Shock is best defined as a life-threatening, generalized form of acute circulatory failure associated with inadequate oxygen utilization by the cells. So, it was adequate to add this concept in the definition of shock because this is the most severe condition in the sepsis progression.^(8)^

Sixth, although there are many limitations in the applicability of the new qSOFA score as will be discussed later, it brought attention to some neglected variables such as reduced level of consciousness and high respiratory rate as markers of disease severity and mortality. But it is only a tool to assess severity and it should not be used to diagnose or define sepsis.^(9)^

Finally, all the controversy generated by the new definitions has brought attention to the sepsis field highlighting the need for further research and investments mainly in educational program and epidemiological studies in low and middle-income areas where there is a paucity of data and shortage of resources.

## The disadvantages

First, the main concern generated by the new definitions is the reduced sensitivity to detect cases that might have an unfavorable course, mainly in low and middle income countries. The new concepts limit the criteria for organ dysfunction and tend to select a more severely ill population.^(10)^ This is a direct consequence of the weight given to predictive validity instead of construct validity used to define sepsis by the task force, which might be of interest in settings where there is excessive sensitivity but is a concern in settings in which patients are not early recognized. In this point of view, efforts on organ dysfunction identification may be more important to not lose opportunities to treat patients, with no change in the treatment approach. This is a new concept approach as no disease in critical care is defined by its ability to predict death, except for acute respiratory distress syndrome. This change in the philosophy behind definitions generates a lot of controversy as we usually seek for improving sensitivity as with the earlier markers to detect acute kidney injury^(11)^ and high sensitivity troponin for myocardial infarction.^(12)^ The use of Sepsis 3 strict criteria for organ dysfunction at bedside might lead to late recognition. For instance, organ dysfunction is defined as a change in 2 points in the SOFA score. As lactate is not part of SOFA score and transient hypotension without vasopressor requirement and Glasgow coma score 13 - 14 scores just 1 point in SOFA, patients with these variables will not fulfill the strict criteria for diagnosis of sepsis. This issue can be minimized as all these conditions are life-threatening organ dysfunction and thus qualify as sepsis as the broad definition of Sepsis 3. In the future, as mentioned in JAMA^(1)^ document, it will be necessary to review organ dysfunction definitions, but one step was done to define sepsis as a severe condition. However, it is important to consider that especially for settings where awareness is already high the gain in specificity of the new definitions can allow private and public institutions to focus their attention and to allocate resources in patients at higher risk of mortality.

Second, the use of variations in SOFA score, even if limited to clinical and epidemiological studies, is not simple. The score is not well known by emergency or ward healthcare professionals and its applicability is complex, as it might demand the calculation of SOFA for subsequent days to verify if the patients fulfill the strict criteria and require laboratory tests. Using SOFA in quality improvement programs to detect sepsis is unfeasible and might delay diagnosis and the starting of antibiotics. The change from the previous severe sepsis criteria (any organ dysfunction) to the new sepsis criteria (variation in SOFA score) was based in a controversial statistical analysis. The authors compared the accuracy of variation in SOFA with the presence of two SIRS criteria, which is not a severity of illness or dysfunction score. Thus, it would not be a surprise to find a better ROC curve for the variation in SOFA. The proposed change in the criteria was not from SIRS to variation in SOFA score. Thus, the relevance in comparing the predictive ability of these criteria is arguable. This is a misleading comparison that apparently would validate the chosen score. The old severe sepsis definition included not only the presence of SIRS but also at least one organ dysfunction. Although it would not be feasible to perform a prediction analysis based on ROC curves, which requires a continuous variable, with a dichotomous variable such as the presence of a single organ dysfunction, other approaches could have been used. Indeed, one among eight patients admitted in ICU with infection associated with one organ dysfunction does not present SIRS criteria;^(6)^ so it could be postulated that suspicious of infection associated with at least one organ dysfunction would be a feasible approach at the bedside and this seems to have a good correlation with severity of illness.^(13)^

A third issue is the devaluation of isolated hyperlactatemia in acute phase of infection as a metabolic organ dysfunction. Although this was not the intention of the task force, the exclusion of lactate as an important marker of cryptic shock might undermine its relevance as a screening laboratory exam to be performed in all patients under suspicion of sepsis. This might compromise the early detection of these severely ill patients, who have high mortality rates. Another potential issue is the bias in epidemiological studies.

A fourth issue is the new definition of septic shock in which hyperlactatemia is a required component for the definition, differently from Sepsis 1 and Sepsis 2 in which just the presence of refractory hypotension to fluid loading was considered shock. The new criteria assume that patients with severe hyperlactatemia but without hypotension have no high risk of death. Although the presence of both variables clearly increases the risk of death, both of them are independent risk factor. Besides of that, as the task force did not point out any other option to lactate as a potential assessment of metabolic abnormalities, the diagnosis of septic shock will be difficult to assess in limited-resource settings where lactate is not available. Although clinical exam can offer a possibility it could not confirm the diagnoses in these setting. Thus, potential shock patients will be considered as having only sepsis, in this scenario it will not possible to estimate precisely septic shock mortality rates.

The fifth issue is the new qSOFA score. We understand that this might be a severity score suitable for identifying patients at high risk of death or ICU stay of more than 3 days in the settings where the data came from. Even in these settings, it was not prospectively validated. However, the authors suggested qSOFA as a screening tool. The statistical model used to select the cut-off point of 2 points aimed to predict morbidity and mortality and not to be a screening tool for early sepsis diagnosis. Therefore, as the authors acknowledged, it needs to be both retrospectively and prospectively assessed and validated before being implemented in our wards and emergency rooms. Initial studies are now showing that qSOFA may have a low sensitivity which is not desirable, but a high specificity to identify patients in a high risk of death, which can just give an alert to approach the patient fastly if this has not yet been done.^(7,14,15)^ In quality improvement programs, our goal is not to identify patients at very high risk of death but rather to identify patients at high risk of deterioration. The usefulness of this score still needs to be determined. It's important to clarify that it is not necessary to wait for two qSOFA criteria to start treatment, and it is just an alert how critically ill the patient is. Waiting for the patient to develop qSOFA criteria to trigger treatment may cause harm.

## Getting a consensus: practical approach to the new definitions

Based on the reasons described above, our major concerns are:

1. The new concepts are being more specific on diagnosis criteria in organ dysfunction, which means that they might reduce sensitivity for critically ill patient if used at bedside and in quality improvement programs.
2. There is a risk of misinterpretation of these new definitions. Sepsis definition is different from infection screening and strategies based on SIRS criteria are still useful in detection of infection. Detection of infection might be the first step to detect a potential septic patient, but the absence of SIRS criteria does not excluded sepsis and it is important to be attentive on patients with suspected infection and any clinical organ dysfunction such as hypotension, low oxygen arterial saturation by pulse oximetry, increased need for oxygen therapy or respiratory support, altered level of consciousness and oliguria.

Considering this and taking into account our main objective to improve sepsis awareness in our country, our proposal for clinical use of the new definitions are:

1. In quality improvement programs, we should use the new broad Sepsis 3.0 definition of sepsis, which is any life-threating organ dysfunction caused by a dysregulated response due to infection. With this broad definition in mind, hypotension, altered level of consciousness and hyperlactatemia are considered life-threatening dysfunctions and those patients require early recognition and treatment. Thus, quality improvement programs should not change their current strategies. This is aligned with the Surviving Sepsis Campaign statement that will continue to use in their quality improvement program the same organ dysfunction criteria, including lactate levels.^(16)^
2. The use of variation in SOFA score as definition of organ dysfunction should be restricted to clinical and epidemiological studies, and should not be used to trigger treatment.
3. The screening for sepsis on patients with suspected infection, both in the emergency department and in the wards, should be based in sensitive tools. Tools based in SIRS criteria or in any clinical (hypotension, reduced level of consciousness, dyspnea, oliguria) or laboratory organ dysfunctions have demonstrated to be of value in several studies.^(17-20)^ The best balance between sensitivity (SIRS) and specificity (organ dysfunction) varies among institutions pending the availability of appropriate resources. Screening for sepsis in the ICU using SIRS criteria is not recommended.
4. The qSOFA should not be used to screen patients for sepsis. It should only be used to identify severe patients with a high risk of death, just as an alert to start sepsis protocol promptly if not already done, when infection is suspected. These patients will need further attention or more close monitoring, if such actions have not already been undertaken.

## Final remarks

In summary, both *Associação de Medicina Intensiva Brasileira* (AMIB) and *Instituto Latino Americano de Sepse* (ILAS) believe that the new broad definition of sepsis, life threatening organ dysfunction due to infection, is adequate. However, both institutions consider that the strict criteria to define organ dysfunction might not be feasible in quality improvement program in our country. We all share the worries about the impact of the new definitions in our settings, in face of our high mortality rates. Some aspects of the new definitions might not be applicable in practical terms at bedside without the risk of a reduction in sensitivity and delay in sepsis recognition.
